# Coronary microthrombi in the failing human heart: the role of von Willebrand factor and PECAM-1

**DOI:** 10.1007/s11010-024-04942-0

**Published:** 2024-02-21

**Authors:** Sawa Kostin, Theodoros Giannakopoulos, Manfred Richter, Florian Krizanic, Benjamin Sasko, Oliver Ritter, Nikolaos Pagonas

**Affiliations:** 1 Faculty of Health Sciences Brandenburg, Brandenburg Medical School Theodor Fontane, Neuruppin, Germany; 2 Department of Internal Medicine and Cardiology, Brandenburg Medical School Theodor Fontane, University Clinic Neuruppin-Brandenburg, Neuruppin, Germany; 3 Department of Cardiac Surgery, Kerckhoff-Clinic, Bad Nauheim, Germany; 4 Medical Department II, Marien Hospital Herne, Ruhr-University of Bochum, Herne, Germany; 5 Department of Cardiology, University Hospital Brandenburg, Brandenburg an der Havel, Germany

**Keywords:** Cardiomyopathies, Microthrombosis, Von Willebrand factor, PECAM-1

## Abstract

The recognition of microthrombi in the heart microcirculation has recently emerged from studies in COVID-19 decedents. The present study investigated the ultrastructure of coronary microthrombi in heart failure (HF) due to cardiomyopathies that are unrelated to COVID-19 infection. In addition, we have investigated the role of von Willebrand factor (VWF) and PECAM-1 in microthrombus formation. We used electron microscopy to investigate the occurrence of microthrombi in patients with HF due to dilated (DCM, *n* = 7), inflammatory (MYO, *n* = 6) and ischemic (ICM, *n* = 7) cardiomyopathy and 4 control patients. VWF and PECAM-1 was studied by quantitative immunohistochemistry and Western blot. In comparison to control, the number of microthrombi was increased 7–9 times in HF. This was associated with a 3.5-fold increase in the number of Weibel–Palade bodies (WPb) in DCM and MYO compared to control. A fivefold increase in WPb in ICM was significantly different from control, DCM and MYO. In Western blot, VWF was increased twofold in DCM and MYO, and more than threefold in ICM. The difference between ICM and DCM and MYO was statistically significant. These results were confirmed by quantitative immunohistochemistry. Compared to control, PECAM-1 was by approximatively threefold increased in all groups of patients. This is the first study to demonstrate the occurrence of microthrombi in the failing human heart. The occurrence of microthrombi is associated with increased expression of VWF and the number of WPb, being more pronounced in ICM. These changes are likely not compensated by increases in PECAM-1 expression.

## Introduction

Cardiomyopathies are a heterogeneous group of heart muscle diseases characterized by structural and functional alterations of the heart [1, 2] and are an important cause of heart failure (HF). Multiple initiating causes and prevailing mechanisms of HF development in several cardiomyopathies have been described [3, 4]. The hallmark of HF due to cardiomyopathies is the structural myocardial remodeling including fibrosis, cardiomyocyte hypertrophy and cardiomyocyte cell death [5–7].

Microvascular spasm has been considered for many decades as a cause of cardiomyopathies and HF [8–11]. While microthrombi formation has been documented in many organs including the brain [12] and kidney [13], the microthrombi in coronary circulation have been largely overlooked. The documentation and recognition of microthrombi in the heart and lung circulation has recently emerged from studies in COVID-19 decedents [14–17].

Recently, Chang proposed two theories of microthrombogenesis [18]. The first theory called inflammatory pathway activation is based on the independent induction of inflammation and microthrombosis in critical illness such as end-stage heart failure. The second theory called microthrombotic pathway activation stems in the damage of blood vessels leading to overexpression/activation of the von Willebrand factor (VWF) and tissue factor [19]. Both theories complement each other and define the thrombogenic mechanisms of fibrin clots and microthrombi [20].

VWF is a large multimeric glycoprotein that mediates initial adhesion of platelets by binding to the platelet GPIIb/IIIa and GPIb receptors [21]. VWF is synthesized mainly in endothelial cells and stored in Weibel–Palade bodies [22]. Upon release from the Weibel–Palade bodies in endothelial cells, VWF in the form of multimers are cleaved by ADAMTS13 (a Distntegrin **a**nd M**e**talloproteinase with a Thrombospondin Type 1 motif, member 13), also known as VWF-cleaving proteinase [23]. VWF multimers are most active in platelet aggregation and failure to cleave large VWF multimers by ADAMTS13 promotes thrombosis [24]. Another important function of VWF is to stabilize the FVIII in the circulation. The VWF-FVIII complex protects FVIII from degradation by thrombin, thereby increasing platelet-platelet and platelet-matrix interaction, leading to fibrin clot formation and thrombosis (reviewed in [25]).

Taken together, VWF has a central role in primary hemostasis where it mediates platelet adhesion to vascular endothelium and subsequently platelet aggregation and thrombus formation.

Platelet endothelial cell adhesion molecule-1 (PECAM-1, CD31) is a 130-kDa membrane glycoprotein that is expressed on vascular endothelial cells and blood cells including platelets, monocytes, neutrophils and lymphocytes [26]. The major functions of PECAM-1 are the regulation of thrombus formation by inhibiting platelet activation pathways [27] and trans-endothelial migration of leukocytes [28]. It has been reported that increased expression and activity of PECAM-1 results in the inhibition of thrombus formation [29, 30].

Given our previous observations that the highest rate of cardiomyocyte cell death has been documented mainly in the immediate vicinity or adjacent to the perivascular area [31, 32], the present study aimed to investigate the ultrastructure and occurrence of coronary microthrombi in the failing human heart due to cardiomyopathies unrelated to COVID-19 infection. Moreover, as mentioned above, the main function of VWF is to initiate platelet adhesion and that PECAM-1 plays an important role in the regulation of the thrombotic process, we have investigated the role of VWF and PECAM-1 as key factors in the microthrombi formation in the coronary circulation in the failing human hearts due to cardiomyopathies.

## Material and methods

### Patients

Human left ventricular (LV) samples were collected from the explanted hearts of patients undergoing orthotopic heart transplantation because of end-stage heart disease. All patients were severely symptomatic (NYHA grade IV) with poor LV systolic function. Patients either had normal coronary arteries with histologically proven chronic myocarditis (MYO, *n* = 6) or without histological evidence of myocardial inflammation (idiopathic dilated cardiomyopathy, DCM, *n* = 7), or had severe coronary artery disease (ischemic cardiomyopathy, ICM, *n* = 7) with a history of previous myocardial infarction (7 of 7). From the latter patients, only the regions distant from myocardial infarctions (remote regions) were investigated. Clinical data are summarized in Table 1. LV myocardium from 3 donor hearts and intraoperative myocardial biopsies from one patient with mitral stenosis but with preserved LV function served as control tissues as previously described [32]. All studies complied with the Declaration of Helsinki and were approved by the ethical committee of the Landesärztekammer Hessen, Frankfurt am Main (project numbers FF 8/2011 and FF 56/2012) and all patients gave written informed consent.

**Table 1:** Clinical characteristics of the patient population

	Control	DCM	MYO	ICM
Number	4	7	6	7
Age	50.4 ± 5.8	49.3 ± 6.7	43.4 ± 4.8*	55.2 ± 2.5
Sex (male/female)	3/1	6/1	4/2	6/1
NYHA class, *n*	–	IV (7/)	IV (6/6)	IV (77)
EF (%)	> 60%	15 ± 2.1	23 ± 4.3	21 ± 2.2
Hypertension	–	1/7	2/6	4/7
Diabetes	–	2/7	3/6	4/7
Chronic pulmonary disease	–	2/7	2/6	2/7
ACE inhibitors (*n*)	1	3	3	3
Beta-blockers (*n*)	1	2	2	2
Ca2 + blockers (*n*)	1	2	2	2
Digitalis (*n*)	–	5	4	5
Diuretics (*n*)	1	6	5	6

*p < 0.5 between ICM and MYO

### Tissue sampling and cells

Myocardial tissues were taken postoperatively from the LV and washed with a cold Krebs–Henseleit solution augmented with 0.1% adenosine and 0.5% albumin. Tissue samples either were immediately frozen for immunohistochemistry and Western blot and stored at − 80 °C, or immersed in 3% glutaraldehyde buffered with 0.1 mmol/L Na cacodylate for electron microscopy.

Human umbilical vein endothelial cells (HUVECs; PromoCell, Heidelberg, Germany) were cultured up to the fourth passage in endothelial cell growth medium (PromoCell, Heidelberg, Germany) supplemented with 5% fetal calf serum (FCS) and gentamicin/amphotericin B. All cell cultures were maintained at 37 °C in a humidified atmosphere with 5% CO_2_ as described [33, 34].

### Immunohistochemistry

The tissue samples were mounted in Tissue-TeK® O.C.T.TM (Sakura) and 5 µm thick cryosections were prepared using a Leica CM3050S cryotome. Before immunolabeling, tissue preservation, characterization and orientation were recorded by hematoxylin and eosin staining. Frozen sections were fixed for 10 min with 4% paraformaldehyde. After washing in phosphate buffered saline (PBS) sections were incubated with 1% bovine serum albumin for 30 min to block non-specific binding sites. and then incubated with the primary antibodies. Monoclonal antibodies against VWF (clone MA5-14,029, ThermoFisher Scientific) and (clone VWF/1465, ab 218,333, Abcam) and a monoclonal antibody against PECAM-1 (clone WM-59, ThermoFisher Scientific) were detected with anti-mouse IgG-conjugated with Cy3 or Cy2 (Biotrend). The nuclei were stained with 1 μg/ml 4′,6-diamidino-2-phenylindole (DAPI, Molecular Probes). F-actin was fluorescently stained using FITC-conjugated (Sigma) or Alexa633-conjugated phalloidin (Molecular Probes). Negative controls were obtained by omitting the primary antibody, in an otherwise similar protocol. Sections were embedded in Mowiol and coverslipped.

### Confocal microscopy

Tissue sections were examined by laser scanning confocal microscopy (Leica TCS SP2 and Leica SP5). Series of confocal optical sections were taken using a Leica Planapo × 40/1.00 or × 63/1.32 objective lens. Each recorded image was taken using four channel scanning and consisted of 1024 × 1024 pixels. To improve image quality and to obtain a high signal to noise ratio, each image from the series was signal-averaged. After data acquisition, the images were further processed for restoration, quantification and three-dimensional reconstruction using an Imaris multichannel image processing software (Bitplane, Zürich, Switzerland).

### Quantitative immunofluorescent microscopy

Measurements of VWF immunofluorescence were done using a × 40 Planapo objective (Leica) and a Leica (Leitz DMRB) fluorescent microscope equipped with a Leica DC380 digital camera. Cryosections from at least two different tissue blocks in each case were used. For quantitative analysis all sections were immunolabeled simultaneously using identical dilutions of primary and secondary antibodies and other reagents. Immunofluorescent images were obtained under identical parameters of imaging, zoom, pinholes, objectives, and fluorescence power. Sections exposed to PBS instead of primary antibodies served as negative controls. The image acquisition settings were standardized for all groups to ensure that the image collected demonstrated a full range of fluorescence intensity from 0 to 255 pixel intensity level and were kept constant during all measurements. Quantification of VWF was performed blindly, having on the screen only one channel showing F-actin labeling. For each patient at least 10 random fields of vision were analyzed using image analysis software (Leica) and Image J program as described [35]. Arbitrary units were calculated per unit surface area (AU/mm^2^).

### Western blot analysis

Tissue samples and cells were processed for Western blot analysis as previously described [35, 36]. In brief, cells and frozen tissues were homogenized in RIPA buffer (containing 20 mmol/L Tris–HCl at pH 7.4, 100 mmol/l NaCl, 5 mmol/L thylene-diamine tetra-acetic acid, 1% Triton ×-100, 10% glycerol, 0.1% sodium dodecylsulfate, 1% deoxycholate, 50 mmol/L NaF, 10 mmol/L Na_4_P_2_O_7_, 1 mmol/l Na_3_VO_4_, 1 mmol/L phenylmethylsulfonylfluoride and mammalian protease inhibitor cocktail (Sigma) at pH 7.4 and centrifuged at 2000×*g* at 4 °C for 10 min. Cellular and LV myocardial extracts were loaded onto 12% polyacrylamide gel and separated under the reducing conditions. Proteins were electro transferred onto nitrocellulose membrane (Invitrogen) and blocked with 5% non-fat dry milk in Tris-buffered saline Tween-20 (TBST) at 4 °C. After washing with TBST, proteins were exposed overnight at 4 °C to monoclonal antibodies against VWF (clone MA5-14,029, ThermoFisher Scientific) and (clone VWF/1465, ab 218,333, Abcam) and a monoclonal antibody against PECAM-1 (clone WM-59, ThermoFisher Scientific) were diluted in TBS with 5% powdered milk. Bound antibodies were detected by peroxidase-conjugated anti-mouse IgG horseradish peroxidase-conjugated and SuperSignal WestFemto (Pierce) detection system and exposed to X-ray film. Quantification of immunoblots was done by scanning on a STORM 860 (Amersham, Pharmacia Biotech) using ImageQuant software as described [36]. The immunoblotting values for the investigated proteins were normalized per beta-actin (Sigma). The control values were set at 100% (see Fig. 4, as an example).

### Transmission electron microscopy

After overnight fixation in 3% glutaraldehyde, small LV tissue samples were embedded in Epon following routine procedures. Ultrathin sections were stained with uranyl acetate and lead citrate, and viewed and photographed in a Philips CM 10 electron microscope. For quantitative ultrastructural analysis of capillaries and endothelial cells, at least 10 fields (300 × 300 μm) per each patient were analyzed.

### Statistical analysis

Results are reported as means ± S.D. Differences between groups were analyzed using ANOVA, followed by secondary analysis by use of Bonferroni’s corrections to Student’s *t*-test. Differences between groups were considered significant at *p* < 0.05.

## Results

### Ultrastructure of microthrombi in cardiomyopathies

**Fig. 1:**
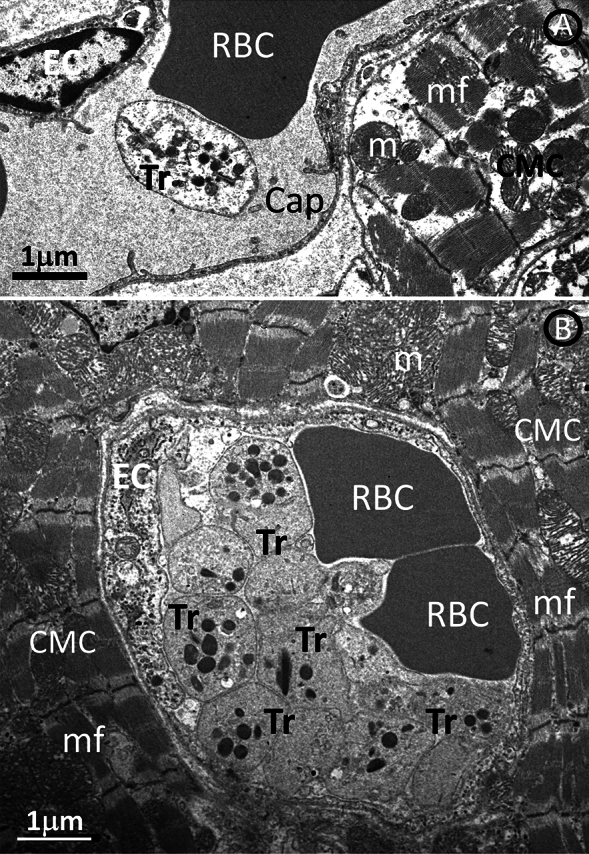
**A** A single platelet in a capillary of a control patient. **B** Intracapillary conglomerates of platelets and erythrocytes in a patient with DCM. Mitochondrial edema and rarefaction of mitochondrial cristae are indicated by arrows. *CMC* cardiomyocyte, *CAP* capillary, *E* erythrocyte, *EC* endotheliocyte, *Tr* platelets, *m* mitochondria, *mf* myofibrils

**Fig. 2:**
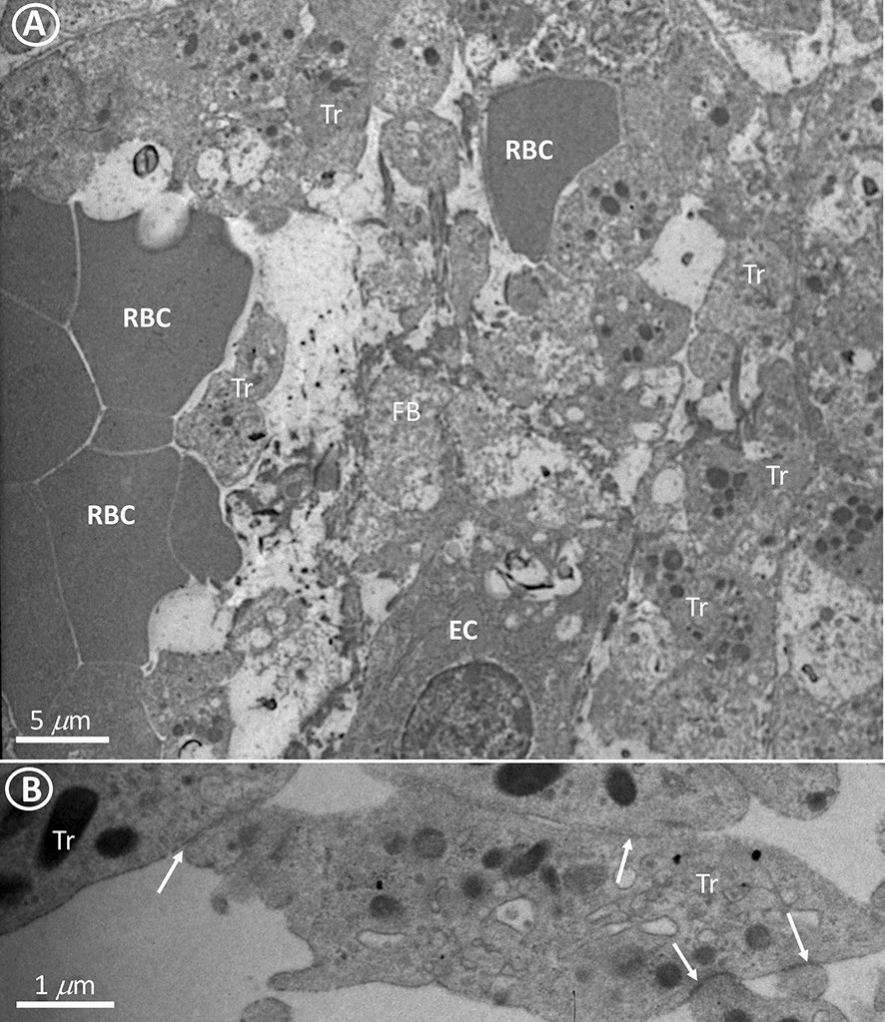
Intracapillary conglomerates of platelets and erythrocytes in a patient with ICM (**A**). **B** is a higher magnification showing that the thrombocytes (Tr) form intercellular adhesions with electron-dense submembranous electron-dense plaques. (arrows), *EC* endothelial cell, *FB* fibrin fibrils, *RBC* red blood cells, *Tr* thrombocytes

**Fig. 3:**
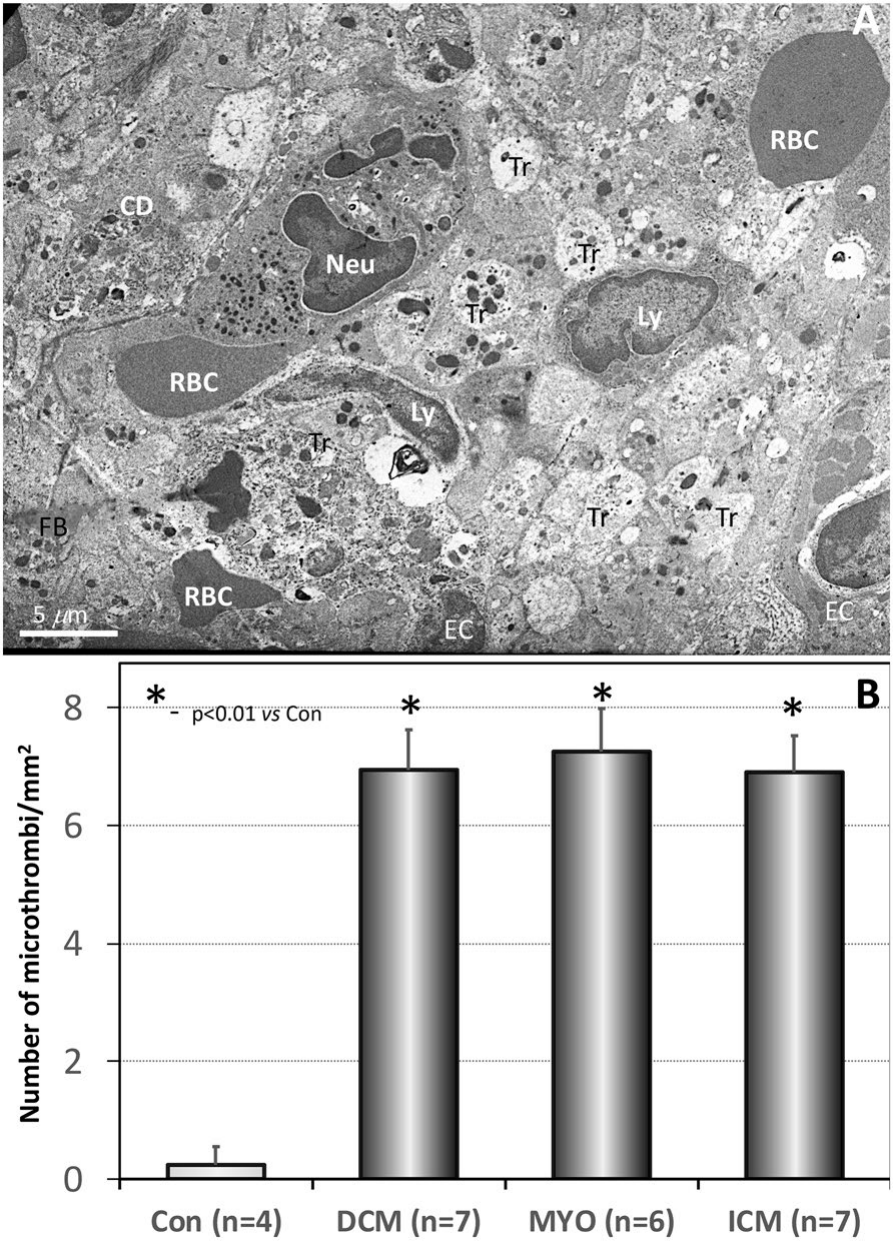
**A** A representative transmission electron image of a thrombus in a patient with MYO characterized by the presence of platelets, red blood cells and inflammatory cells including lymphocytes and neutrophils. *CD* cellular debris, *Neu* neutrophil, *Ly* lymphocytes, *RBC* red blood cells, *EC* endotheliocyte, *Tr* thrombocytes. **B** Quantification of microthrombi in the studied groups

We used electron microscopy to detect microthrombi. Figure 1 compares the ultrastructure of a capillary in a control patient and a capillary in a patient with DCM only a single, solitary platelet is observed intracapillary in a control patient (Fig. 1A). In DCM, platelets adhered firmly to each other and to endothelial cells and form conglomerates with erythrocytes (Fig. 1B). Figure 2A is a representative electron microscopic image of a microthrombus in a patient with ICM. Figure 2B is a higher magnification and shows that the platelets form intercellular adhesions with electron-dense submembranous electron-dense plaques that exclude post-fixation artifacts. Compared to microthrombi seen in patients with DCM and ICM, microthrombi in MYO patients were characterized not only by aggregates of platelets and red blood cells, but also by the presence of inflammatory cells, including lymphocytes and neutrophils (Fig. 3A). Figure 3B shows the quantification of the number of microthrombi per myocardial surface and shows a sevenfold increase in the number of microthrombi in cardiomyopathic hearts compared to control hearts.

### Von Willebrand factor is drastically increased in cardiomyopathies

**Fig. 4:**
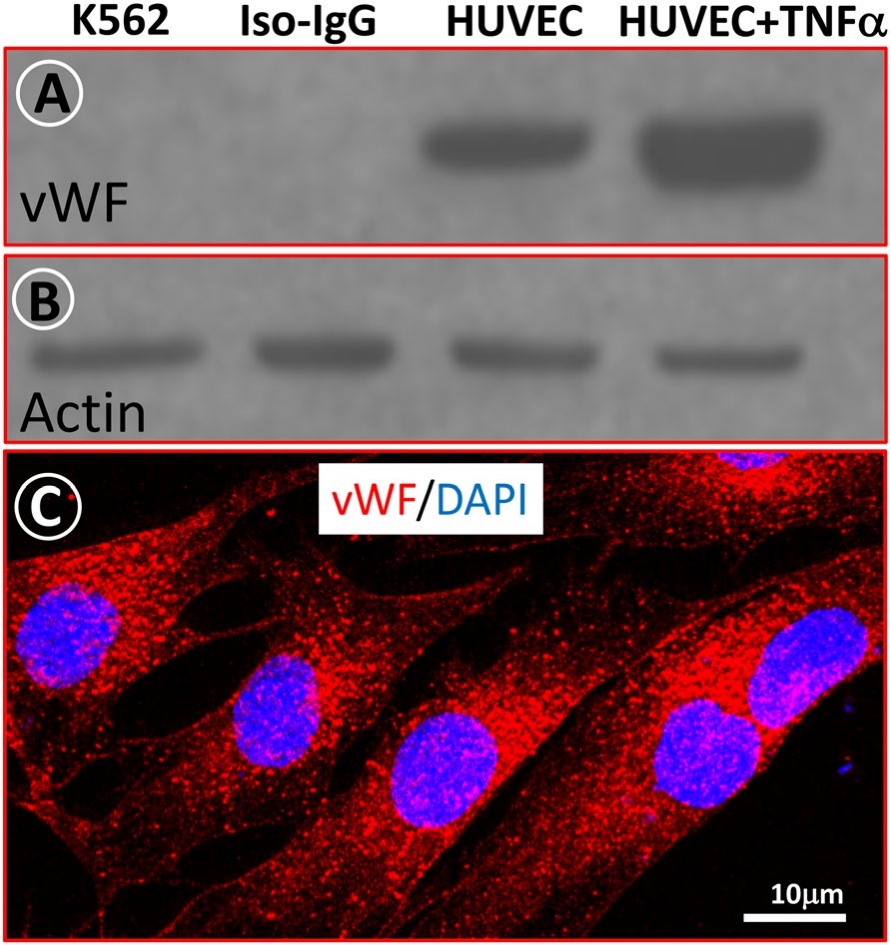
Expression of VWF in cultured HUVECs. **A** representative Western blot image of VWF in K562 leukemia cells and HUVECs. Note the absence of VWF in K562 cells or when replacing the primary antibody with IgG of the same species (isotypic IgG). Also notice the increase in VWF in HUVECs after stimulation of these cells with TNFα. **B** Western blot image using the same blot membrane as in panel A, incubated with antibodies against beta-actin. **C** Confocal microscopy of HUVECs. VWF is labeled in red with Cy3, and nuclei are stained blue with DAPI. Note the granular pattern of the VWF fluorescent signal

To study VWF at the protein level, it was necessary to validate monoclonal antibodies in protein extracts of human leukemia cells K562 (negative cell control) and HUVEC, with or without stimulation with the pro-inflammatory cytokine TNF-alpha (positive cell control). Figure 4A and B show representative immunoblots and quantification of VWF (52 kDA) in the cell extracts studied and demonstrates that this factor is absent in K562 cell and in blotting membranes incubated with isotypic IgG. It is important to note that the level of VWF is obviously more pronounced in HUVECs exposed to TNFα than in unstimulated HUVECs.

In addition, antibodies against VWF were tested by immunohistochemistry and confocal microscopy. Figure 4C is a representative image of HUVEC showing a granular cytoplasmic pattern of fluorescent VWF (52 kDa) signal. In addition, we tested by immunohistochemistry a monoclonal antibody against high-molecular weight of VWF (250 kDa) in HUVEC cultures (data not shown).

**Fig. 5:**
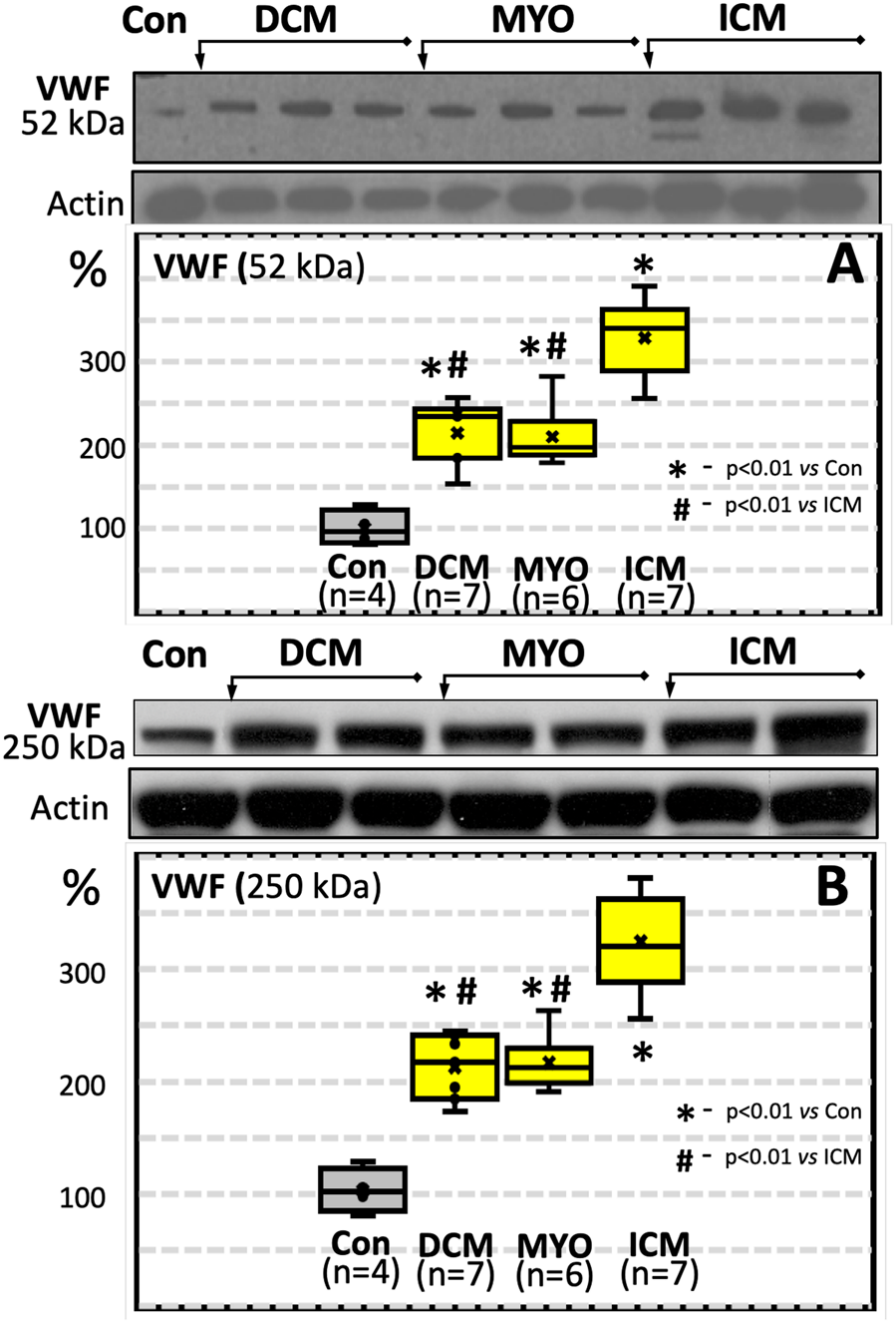
Representative Western blot gels and boxplots of densitometric quantification of 52 kDA VWF (**A**) and of 250 kDa VWF (**B**) in human myocardium

The expression of VWF in the control group and in cardiomyopathies was studied at the protein level. Figure 5A shows representative immunoblots for VWF (52 kDA) and their quantitative data. Compared to the control group, the VWF level is significantly increased in all patients with cardiomyopathies. It is noteworthy that VWF is not only significantly increased in ICM compared to the control group (*p* < 0.001), but also compared to the DCM (*p* = 0.0286) and MYO (*p* = 0.0349) groups. Similar results were documented in immunoblots for high-molecular weight (250 kDA) VWF (Fig. 5B).

**Fig. 6:**
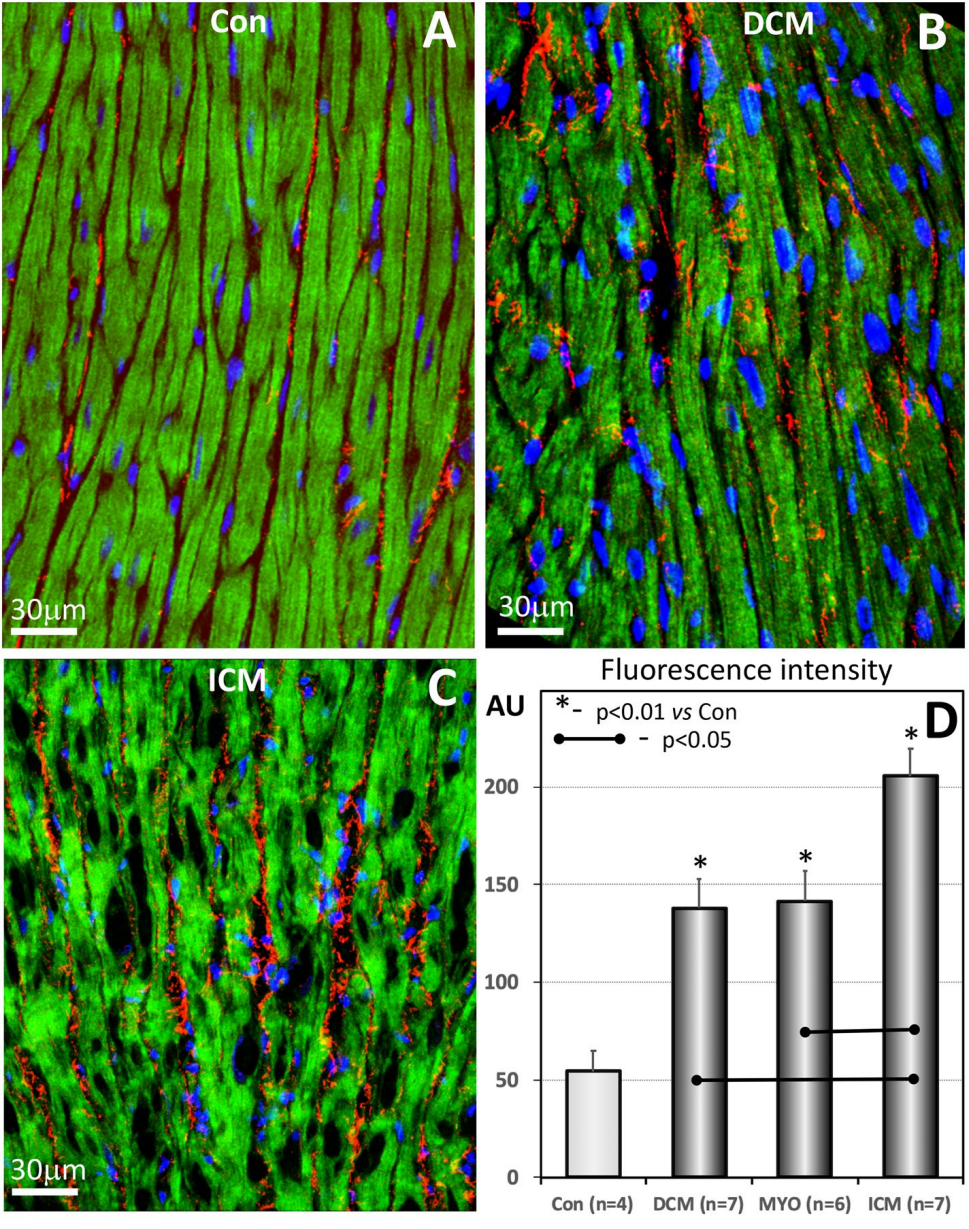
Representative confocal images of VWF expression in a control (**A**), in a DCM (**B**) and in an ICM patient (**C**). Scale bar 30 μm. Quantification of VWF is shown in **D**

Confocal microscopy and quantitative immunohistochemistry using antibodies against VWF showed that VWF signal is significantly higher in all patients with cardiomyopathies compared to controls (Fig. 6).

### Weibel–Palade bodies are significantly increased in cardiomyopathies

**Fig. 7:**
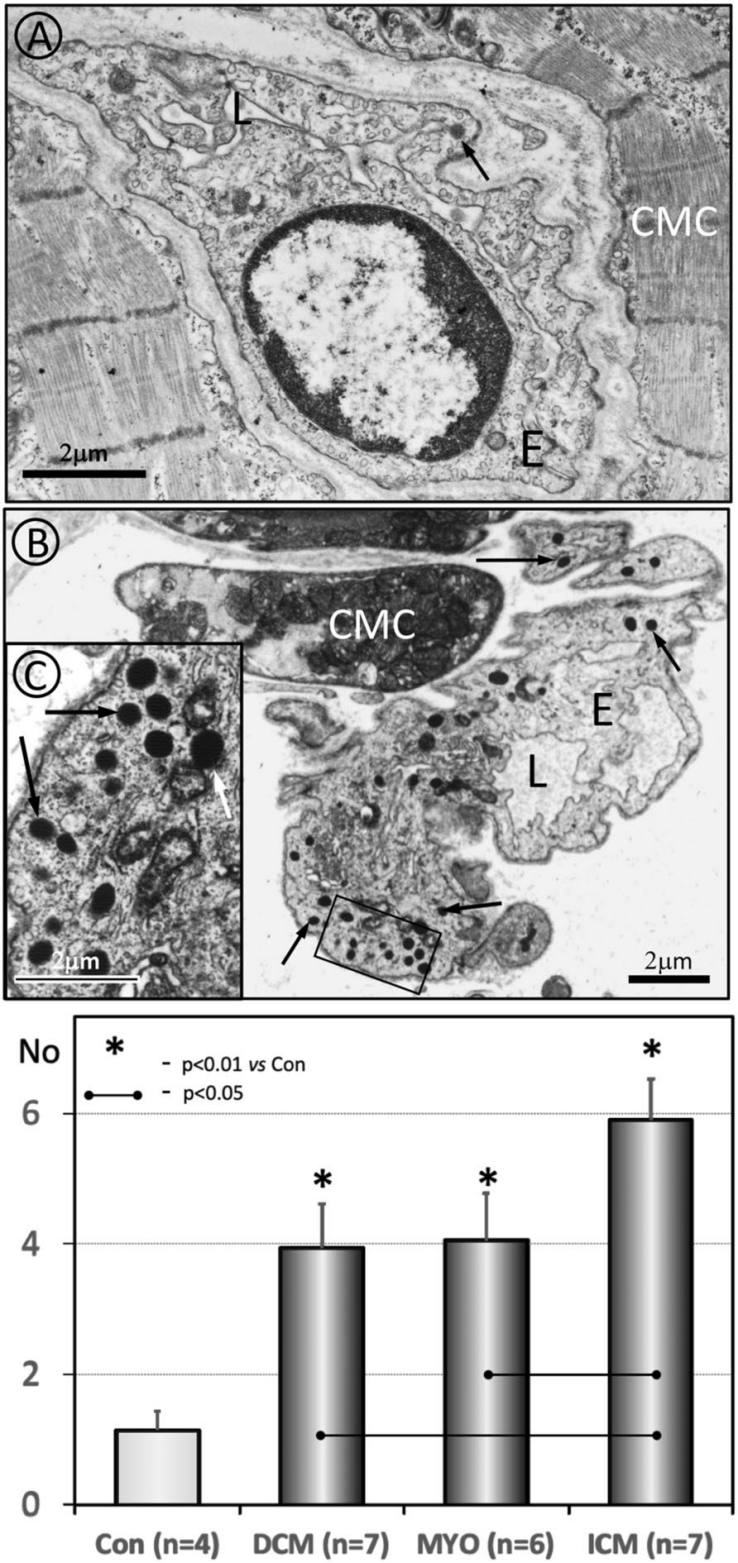
Electron microscopy images of endothelial cells in normal myocardium (**A**) and in a patient with ICM (**B**). Weibel–Palade bodies are marked with arrows in both panels. Note the increased number of Weibel–Palade bodies in the endothelium of the patient with ICM. Also worth mentioning is the significant increase in the electron-optical density of these structures compared to those of the control group. **C** is a higher magnification of the marked area in **B**. *CMC* cardiomyocyte, *E* endothelium, *L* lumen. **D** Average number of Weibel–Palade bodies per 100 square micrometers of endothelium in the human myocardium in control, DCM, MYO and ICM patients

It is well known that Weibel–Palade granules (bodies) in endothelial cells are the main source of VWF. In this study, Weibel–Palade bodies in endothelial cells were investigated using electron microscopy. Figure 7A and B compares Weibel–Palade bodies in endothelial cells from a control myocardium with those from a remote myocardium from a patient with ICM. It is evident that in ICM the Weibel–Palade bodies are drastically increased in number and size. Moreover, the Weibel–Palade bodies in the latter case are electron-dense, which suggests their maturity, since more lucidity observed in the control endothelium indicates earlier stages of their formation.

As a next step, we quantified the number of Weibel–Palade bodies in the studied groups. The data are presented in Fig. 7C. It was found that 100 square micrometers of endothelial surface area in the control group comprised an average of 1.14 ± 0.3 Weibel–Palade bodies. The same endothelial surface area comprised 3.93 ± 0.68 Weibel–Palade bodies in DCM, 4.04 ± 0.73 in MYO and 5.93 ± 0.62 in ICM. It should be emphasized that the values of the latter group differed significantly from those of the DCM group (*p* = 0.012) and the MYO group (*p* = 0.019).

### PECAM-1 is increased in cardiomyopathies

**Fig. 8:**
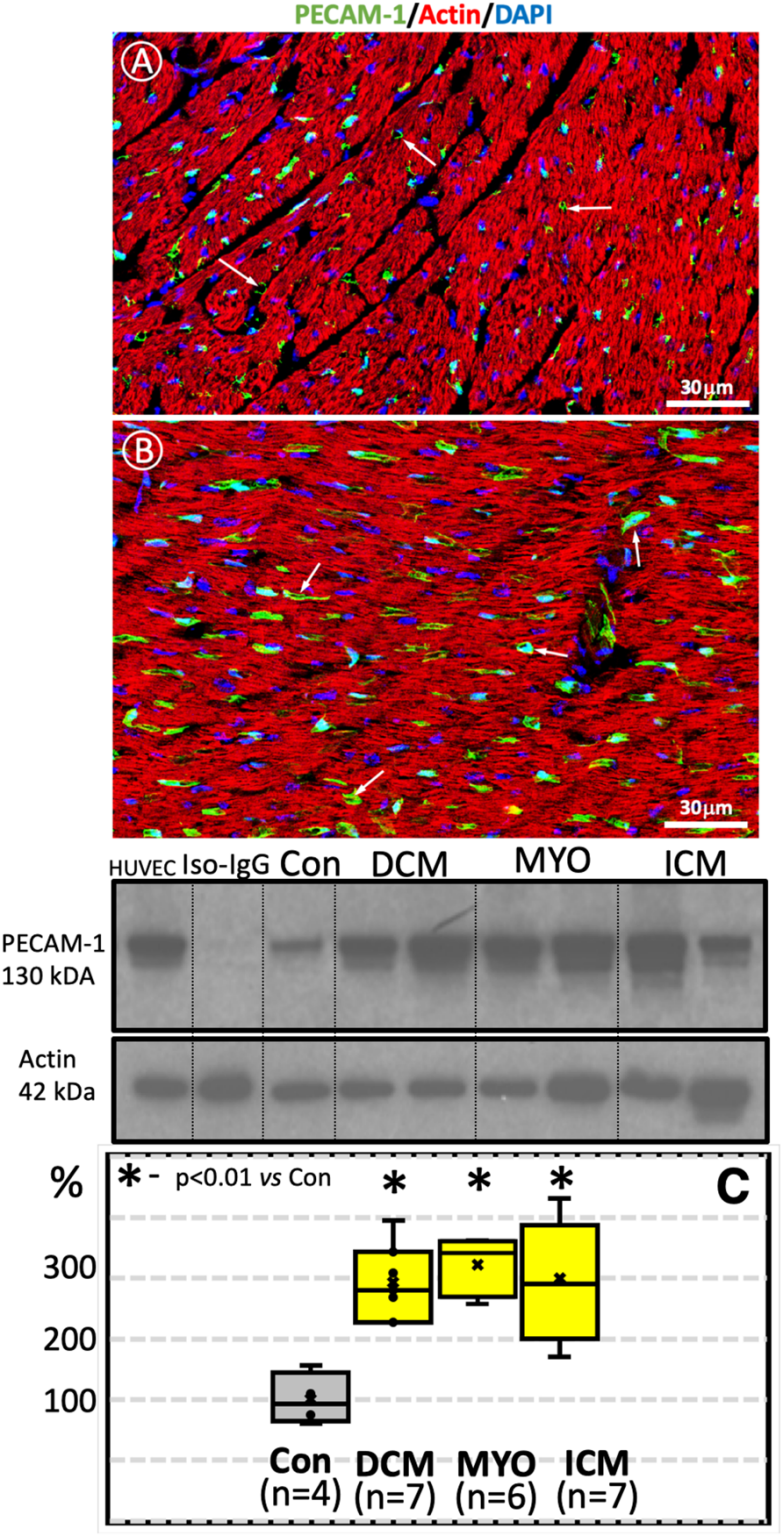
PECAM-1 in human myocardium from a control (**A**), and a DCM patient (**B**). Note the vascular distribution pattern (Cy2-green, arrows) in both cases. Note an obvious increase in the PECAM-1 signal in DCM. Both images were acquired with the same laser power and the same background level of the confocal microscope. F-actin is labeled in red with Phalloidin-633, and nuclei are stained in blue with DAPI. **C:** Representative Western blot images of PECAM-1 and their quantification. HUVEC served as positive controls and express PECAM-1 protein with a specific molecular weight of 130 kDa. For the quantification of PECAM-1 in the cardiomyopathic groups, the values of this protein were set to 100% of the values in the control group

The immunohistochemical study with monoclonal antibodies, fluorescent conjugates and confocal microscopy revealed a vascular distribution pattern of PECAM-1 in the human myocardium in all investigated groups (Fig. 8A and B).

The expression of PECAM-1 in the myocardium of patients with cardiomyopathies was studied by Western immunoblotting. HUVEC from passage 4 in culture were used as a positive control. Blot membranes incubated with isotypic IgG instead of primary antibodies were used as a negative control. The amount of PECAM 1 was calculated by densitometry and correlated with that of beta-actin. Figure 8C shows representative immunoblots of PECAM-1 expression in the analyzed material. Quantitative analysis of immunoblots showed that PECAM-1 increased 3.16-fold in DCM patients, 3.4-fold in MYO patients and 2.97-fold in patients with ICM compared to the control group (Fig. 8C).

## Discussion

In this study, the occurrence of microthrombi in the coronary microcirculation of the failing human heart is demonstrated for the first time. We show that thrombus formation is associated with a drastically increased expression of VWF and the number of Weibel–Palade bodies. Interestingly, a threefold increased PECAM-1 cannot prevent thrombus formation, however it might attract inflammatory cells [28] that are commonly observed in the failing human heart [37].

In the development of HF due to cardiomyopathies, regardless of etiology, extensive remodeling of the entire myocardium occurs and includes cardiomyocyte degeneration and death, macrophage and fibroblast activation, cytokine storm, replacement and perivascular fibrosis leading to reduced cardiac function and thus establishing a vicious circle [3, 38, 39]. In this context, the pathophysiological consequence of the occurrence of coronary microthrombosis in HF, documented in the present study, is a part of this vicious circle as an important contributor to inadequate myocardial perfusion and dysfunction.

The role of myocardial microthrombi in cardiac pathology has recently emerged from studies recently carried out in autopsy material in COVID-19 decedents [14–17]. In comparison with our data in the failing human heart due to cardiomyopathies, coronary vascular microthrombi in COVID-19 patients is not dependent on endothelial cell activation assessed by expression of endothelial adhesion molecules or VWF. The main player in COVID-19-induced microthrombi are activated circulating neutrophils, which have been regularly observed in the thrombi of COVID-19 patients [16].

The main function of VWF is to initiate platelet adhesion upon vascular injury [40]. The hallmark of acute and chronic inflammation is the widespread activation of endothelial cells, which provokes excessive VWF secretion from the endothelial cell storage pool [34]. The VWF level in blood reflects the early state of endothelial activation, and the increase in VWF blood level is either due to a pathological increase in the rate of basal VWF secretion or is increased by dysfunctional and/or activated endothelial cells [40]. The increase in plasma VWF is predictive of prothrombotic complications and multi-organ system failure associated with reduced survival in the context of severe inflammatory response syndrome, type II diabetes mellitus, stroke and in diverse cardiac diseases [41, 42]. Pertinent to our study are the recent observations that elevated VWF levels are associated with HF with preserved ejection fraction and may serve as a potential biomarker of HF severity [43].

Our study demonstrating increased expression and deposition of VWF in Weibel–Palade bodies at the cellular level has clinical implications in VWF-related drug development strategies at the capillary levels. Thus, it has been demonstrated that simvastatin administration alleviates microthrombosis in a rat model of subarachnoid hemorrhage [44]. Moreover, recent findings suggest that the use of histamine-H1R antagonists may prevent cardiac microthrombosis [45].

## Conclusions

This is the first study to demonstrate the microthrombi in the failing human heart. The occurrence of microthrombi is associated with increased expression of VWF and the number of Weibel–Palade bodies and is not compensated by increases in PECAM-1 expression.

## Data Availability

Enquiries about data availability should be directed to the authors.

## References

[1] Maron BJ, Towbin JA, Thiene G, Antzelevitch C, Corrado D, Arnett D, Moss AJ, Seidman CE, Young JB, American Heart A et al (2006) Contemporary definitions and classification of the cardiomyopathies: an American heart association scientific statement from the council on clinical cardiology, heart failure and transplantation committee; quality of care and outcomes research and functional genomics and translational biology interdisciplinary working groups; and council on epidemiology and prevention. Circulation 113(14):1807–1816 16567565 10.1161/CIRCULATIONAHA.106.174287

[2] Seferovic PM, Polovina M, Bauersachs J, Arad M, Ben Gal T, Lund LH, Felix SB, Arbustini E, Caforio ALP, Farmakis D et al (2019) Heart failure in cardiomyopathies: a position paper from the heart failure association of the European society of cardiology. Eur J Heart Fail 21(5):553–576 30989768 10.1002/ejhf.1461

[3] Cohn JN, Ferrari R, Sharpe N (2000) Cardiac remodelling–concepts and clinical implications: a consensus paper from an international forum on cardiac remodelling. Behalf of an international forum on cardiac remodeling. J Am Coll Cardiol 35(3):569–582 10716457 10.1016/s0735-1097(99)00630-0

[4] Braunwald E (2017) Cardiomyopathies: an overview. Circ Res 121(7):711–721 28912178 10.1161/CIRCRESAHA.117.311812

[5] Brilla CG, Maisch B (1994) Regulation of the structural remodelling of the myocardium: from hypertrophy to heart failure. Eur Heart J 15:45–52 7713113 10.1093/eurheartj/15.suppl_d.45

[6] Kostin S (2011) Types of cardiomyocyte death and clinical outcomes in patients with heart failure. J Am Coll Cardiol 57(14):1532–1534 21453831 10.1016/j.jacc.2010.10.049

[7] Arbustini E, Narula N, Tavazzi L, Serio A, Grasso M, Favalli V, Bellazzi R, Tajik JA, Bonow RO, Fuster V et al (2014) The MOGE(S) classification of cardiomyopathy for clinicians. J Am Coll Cardiol 64(3):304–318 25034069 10.1016/j.jacc.2014.05.027

[8] Sonnenblick EH, Fein F, Capasso JM, Factor SM (1985) Microvascular spasm as a cause of cardiomyopathies and the calcium-blocking agent verapamil as potential primary therapy. Am J Cardiol 55(3):179–184 10.1016/0002-9149(85)90629-0 3881912

[9] Figulla H, Vetterlein F, Glaubitz M, Kreuzer H (1987) Inhomogenous capillary flow and its prevention by verapamil and hydralazine in the cardiomyopathic Syrian hamster. Circulation 76:208–216 2439233 10.1161/01.cir.76.1.208

[10] Ramos SG, Soares FA, Bestetti RB, Samuel JMO, Mello-Oliveiraq JA, Rossi MA (1996) Cardiomyopathy in rats with Walker 256 tumor: the potential role of microvascular disease in its genesis. Cardiovasc Pathol 5(1):39–46 25851211 10.1016/1054-8807(95)00032-1

[11] Wheeler MT, Korcarz CE, Collins KA, Lapidos KA, Hack AA, Lyons MR, Zarnegar S, Earley JU, Lang RM, McNally EM (2004) Secondary coronary artery vasospasm promotes cardiomyopathy progression. Am J Pathol 164(3):1063–1071 14982859 10.1016/S0002-9440(10)63193-8 PMC1614719

[12] Kawai H, Umemura K, Nakashima M (1995) Effect of argatroban on microthrombi formation and brain damage in the rat middle cerebral artery thrombosis model. Jpn J Pharmacol 69(2):143–148 8569051 10.1254/jjp.69.143

[13] Galindo M, Gonzalo E, Martinez-Vidal MP, Montes S, Redondo N, Santiago B, Loza E, Pablos JL (2009) Immunohistochemical detection of intravascular platelet microthrombi in patients with lupus nephritis and anti-phospholipid antibodies. Rheumatology (Oxford) 48(8):1003–1007 19542214 10.1093/rheumatology/kep152

[14] Guagliumi G, Sonzogni A, Pescetelli I, Pellegrini D, Finn AV (2020) Microthrombi and ST-segment-elevation myocardial infarction in COVID-19. Circulation 142(8):804–809 32677840 10.1161/CIRCULATIONAHA.120.049294 PMC7439756

[15] Ackermann M, Verleden SE, Kuehnel M, Haverich A, Welte T, Laenger F, Vanstapel A, Werlein C, Stark H, Tzankov A et al (2020) Pulmonary vascular endothelialitis, thrombosis, and angiogenesis in Covid-19. N Engl J Med 383(2):120–128 32437596 10.1056/NEJMoa2015432 PMC7412750

[16] Johnson JE, McGuone D, Xu ML, Jane-Wit D, Mitchell RN, Libby P, Pober JS (2022) Coronavirus disease 2019 (COVID-19) coronary vascular thrombosis: correlation with neutrophil but not endothelial activation. Am J Pathol 192(1):112–120 34599881 10.1016/j.ajpath.2021.09.004 PMC8479934

[17] Brener MI, Hulke ML, Fukuma N, Golob S, Zilinyi RS, Zhou Z, Tzimas C, Russo I, McGroder C, Pfeiffer RD et al (2022) Clinico-histopathologic and single-nuclei RNA-sequencing insights into cardiac injury and microthrombi in critical COVID-19. JCI Insight. 10.1172/jci.insight.154633 34905515 10.1172/jci.insight.154633 PMC8855793

[18] Chang JC (2018) Hemostasis based on a novel ‘two-path unifying theory’ and classification of hemostatic disorders. Blood Coagul Fibrinol 29(7):573–584 10.1097/MBC.0000000000000765 30063477

[19] Chang JC (2018) Thrombogenesis and thrombotic disorders based on ‘two-path unifying theory of hemostasis’: philosophical, physiological, and phenotypical interpretation. Blood Coagul Fibrinol 29(7):585–595 10.1097/MBC.0000000000000769 30234545

[20] Chang JC (2020) Stroke classification: critical role of unusually large von Willebrand factor multimers and tissue factor on clinical phenotypes based on novel “two-path unifying theory” of hemostasis. Clin Appl Thromb Hemost 26:1076029620913634 32584600 10.1177/1076029620913634 PMC7427029

[21] Ruggeri ZM (2003) Von Willebrand factor, platelets and endothelial cell interactions. J Thromb Haemost 1(7):1335–1342 12871266 10.1046/j.1538-7836.2003.00260.x

[22] Michaux G, Abbitt KB, Collinson LM, Haberichter SL, Norman KE, Cutler DF (2006) The physiological function of von Willebrand’s factor depends on its tubular storage in endothelial Weibel–Palade bodies. Dev Cell 10(2):223–232 16459301 10.1016/j.devcel.2005.12.012

[23] Levy GG, Motto DG, Ginsburg D (2005) ADAMTS13 turns 3. Blood 106(1):11–17 15774620 10.1182/blood-2004-10-4097

[24] Dong JF, Moake JL, Nolasco L, Bernardo A, Arceneaux W, Shrimpton CN, Schade AJ, McIntire LV, Fujikawa K, Lopez JA (2002) ADAMTS-13 rapidly cleaves newly secreted ultralarge von Willebrand factor multimers on the endothelial surface under flowing conditions. Blood 100(12):4033–4039 12393397 10.1182/blood-2002-05-1401

[25] Terraube V, O’Donnell JS, Jenkins PV (2010) Factor VIII and von Willebrand factor interaction: biological, clinical and therapeutic importance. Haemophilia 16(1):3–13 19473409 10.1111/j.1365-2516.2009.02005.x

[26] Albelda SM, Muller WA, Buck CA, Newman PJ (1991) Molecular and cellular properties of PECAM-1 (endoCAM/CD31): a novel vascular cell-cell adhesion molecule. J Cell Biol 114(5):1059–1068 1874786 10.1083/jcb.114.5.1059 PMC2289123

[27] Soriano Jerez EM, Gibbins JM, Hughes CE (2021) Targeting platelet inhibition receptors for novel therapies: PECAM-1 and G6b-B. Platelets 32(6):761–769 33646086 10.1080/09537104.2021.1882668

[28] Muller WA, Weigl SA, Deng X, Phillips D (1993) PECAM-1 is required for transendothelial migration of leukocytes. J Exp Med 178:449–460 8340753 10.1084/jem.178.2.449 PMC2191108

[29] Jones KL, Hughan SC, Dopheide SM, Farndale RW, Jackson SP, Jackson DE (2001) Platelet endothelial cell adhesion molecule-1 is a negative regulator of platelet-collagen interactions. Blood 98(5):1456–1463 11520795 10.1182/blood.v98.5.1456

[30] Falati S, Patil S, Gross PL, Stapleton M, Merrill-Skoloff G, Barrett NE, Pixton KL, Weiler H, Cooley B, Newman DK et al (2006) Platelet PECAM-1 inhibits thrombus formation in vivo. Blood 107(2):535–541 16166583 10.1182/blood-2005-04-1512 PMC1895610

[31] Kostin S (2005) Pathways of myocyte death: implications for development of clinical laboratory biomarkers. Adv Clin Chem 40:37–98 16355920 10.1016/s0065-2423(05)40002-5

[32] Kostin S, Pool L, Elsasser A, Hein S, Drexler HC, Arnon E, Hayakawa Y, Zimmermann R, Bauer E, Klovekorn WP et al (2003) Myocytes die by multiple mechanisms in failing human hearts. Circ Res 92(7):715–724 12649263 10.1161/01.RES.0000067471.95890.5C

[33] Kubin T, Ando H, Scholz D, Bramlage P, Kostin S, van Veen A, Heling A, Hein S, Fischer S, Breier A et al (1999) Microvascular endothelial cells remodel cultured adult cardiomyocytes and increase their survival. Am J Physiol 276(6):H2179-2187 10362702 10.1152/ajpheart.1999.276.6.H2179

[34] Dmitrieva NI, Burg MB (2014) Secretion of von Willebrand factor by endothelial cells links sodium to hypercoagulability and thrombosis. Proc Natl Acad Sci USA 111(17):6485–6490 24733925 10.1073/pnas.1404809111 PMC4035979

[35] Petersen F, Rodrigo R, Richter M, Kostin S (2017) The effects of polyunsaturated fatty acids and antioxidant vitamins on atrial oxidative stress, nitrotyrosine residues, and connexins following extracorporeal circulation in patients undergoing cardiac surgery. Mol Cell Biochem 433(1–2):27–40 28337705 10.1007/s11010-017-3013-1

[36] Polyakova V, Loeffler I, Hein S, Miyagawa S, Piotrowska I, Dammer S, Risteli J, Schaper J, Kostin S (2011) Fibrosis in endstage human heart failure: severe changes in collagen metabolism and MMP/TIMP profiles. Int J Cardiol 151(1):18–33 20546954 10.1016/j.ijcard.2010.04.053

[37] Devaux B, Scholz D, Hirche A, Klovekorn WP, Schaper J (1997) Upregulation of cell adhesion molecules and the presence of low grade inflammation in human chronic heart failure. Eur Heart J 18(3):470–479 9076385 10.1093/oxfordjournals.eurheartj.a015268

[38] Elsässer A, Kostin S, Hein S, Zimmermann R, Schaper J (2001) Remodeling and cell death in failing human myocardium. Coronary Artery Dis 7:19–24

[39] Schaper J, Kostin S, Hein S, Elsasser A, Arnon E, Zimmermann R (2002) Structural remodelling in heart failure. Exp Clin Cardiol 7(2–3):64–68 19649225 PMC2719171

[40] van Nispen tot Pannerden H, de Haas F, Geerts W, Posthuma G, van Dijk S, Heijnen HF (2010) The platelet interior revisited: electron tomography reveals tubular alpha-granule subtypes. Blood 116(7):1147–1156 20439620 10.1182/blood-2010-02-268680

[41] Frankel DS, Meigs JB, Massaro JM, Wilson PW, O’Donnell CJ, D’Agostino RB, Tofler GH (2008) Von Willebrand factor, type 2 diabetes mellitus, and risk of cardiovascular disease: the framingham offspring study. Circulation 118(24):2533–2539 19029465 10.1161/CIRCULATIONAHA.108.792986 PMC2746947

[42] Spiel AO, Gilbert JC, Jilma B (2008) von Willebrand factor in cardiovascular disease: focus on acute coronary syndromes. Circulation 117(11):1449–1459 18347221 10.1161/CIRCULATIONAHA.107.722827

[43] Abudoukelimu M, Ba B, Kai Guo Y, Xu J (2022) Von Willebrand factor (vWF) in patients with heart failure with preserved ejection fraction (HFpEF): a retrospective observational study. Medicine (Baltimore) 101(31):e29854 35945712 10.1097/MD.0000000000029854 PMC9351886

[44] Wang Z, Chen G, Zhu WW, Bian JY, Shen XO, Zhou D (2010) Influence of simvastatin on microthrombosis in the brain after subarachnoid hemorrhage in rats: a preliminary study. Ann Clin Lab Sci 40(1):32–42 20124328

[45] Yang X, Shi Z, Wang X, Yang Y, Sun D, Zhu B, Song F, Zhu X, Ding S, Zou Y et al (2023) Disruption of histamine-H(1)R signaling exacerbates cardiac microthrombosis after periodontal disease via TLR4/NFkappaB-p65 pathway. Int Immunopharmacol 123:110774 37567012 10.1016/j.intimp.2023.110774

